# Atrial septal perforation with Needle's Eye Snare during transvenous lead extraction

**DOI:** 10.1002/joa3.12593

**Published:** 2021-07-11

**Authors:** Tsuyoshi Isawa, Taku Honda, Kazuhiro Yamaya, Yusuke Hasegawa, Jun Ito

**Affiliations:** ^1^ Department of Cardiology Sendai Kousei Hospital Sendai Japan; ^2^ Department of Cardiovascular Surgery Sendai Kousei Hospital Sendai Japan; ^3^ Department of Anesthesiology and Intensive Care Unit Sendai Kousei Hospital Sendai Japan

**Keywords:** atrial septal perforation, coronary air embolism, femoral approach, lead extraction, Needle's Eye Snare

## Abstract

Although the Needle's Eye Snare (Cook Medical) has been considered useful for lead extraction, serious complications can occur. We presented a case of atrial septal perforation associated with the Needle's Eye Snare. Our case highlights the importance of not persisting with the Needle's Eye Snare to prevent atrial damage.

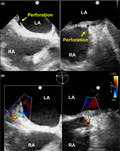

## 1 INTRODUCTION

The Needle's Eye Snare (Cook Medical) is a useful tool for transfemoral lead extraction. It has the advantage of being able to grasp a lead without a free end and pull it caudally, which makes the advancement of a powered sheath over the targeted lead via the implant vein much easier in a combined superior and femoral approach. However, serious complications can occur when using this device. Herein, we describe a case of a coronary air embolism secondary to an atrial septal perforation.

## 2 CASE PRESENTATION

**FIGURE 1:**
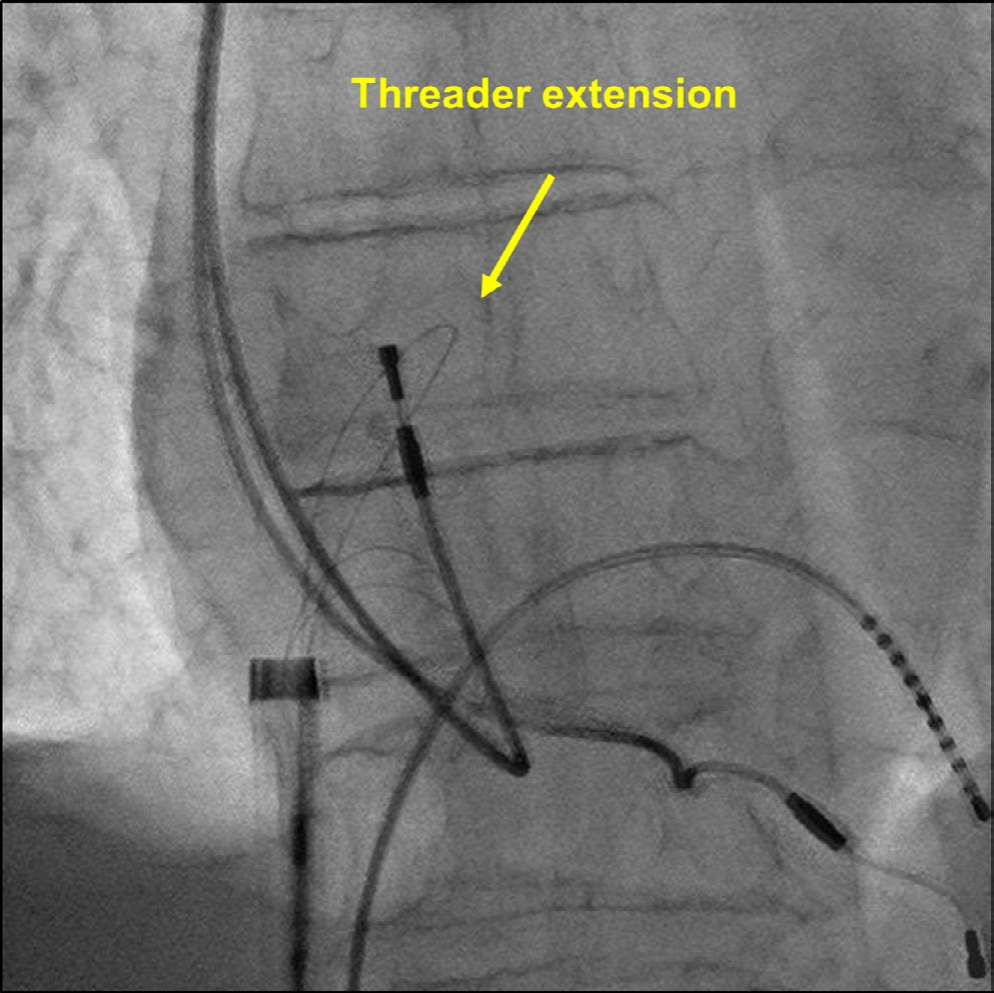
Needle's Eye Snare and threader extended between contralateral snare struts

**FIGURE 2:**
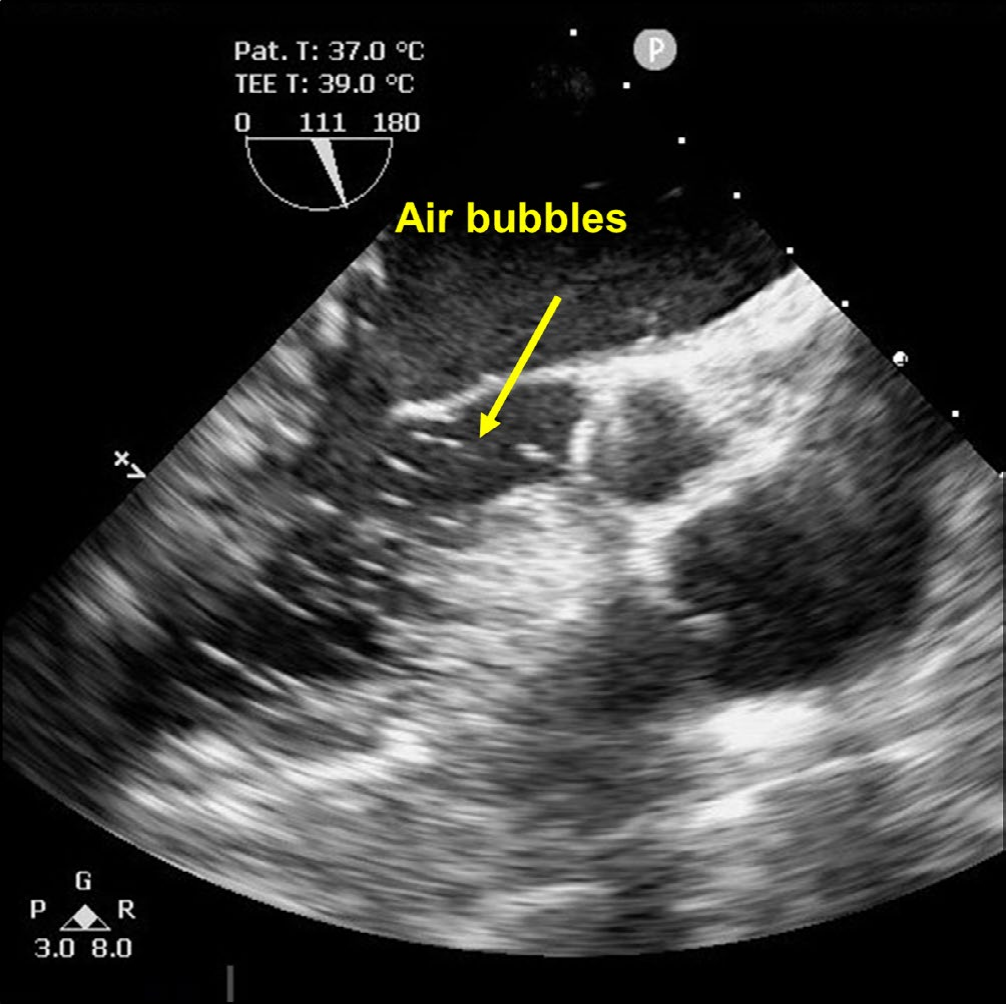
Air bubbles in the left ventricle

**FIGURE 3:**
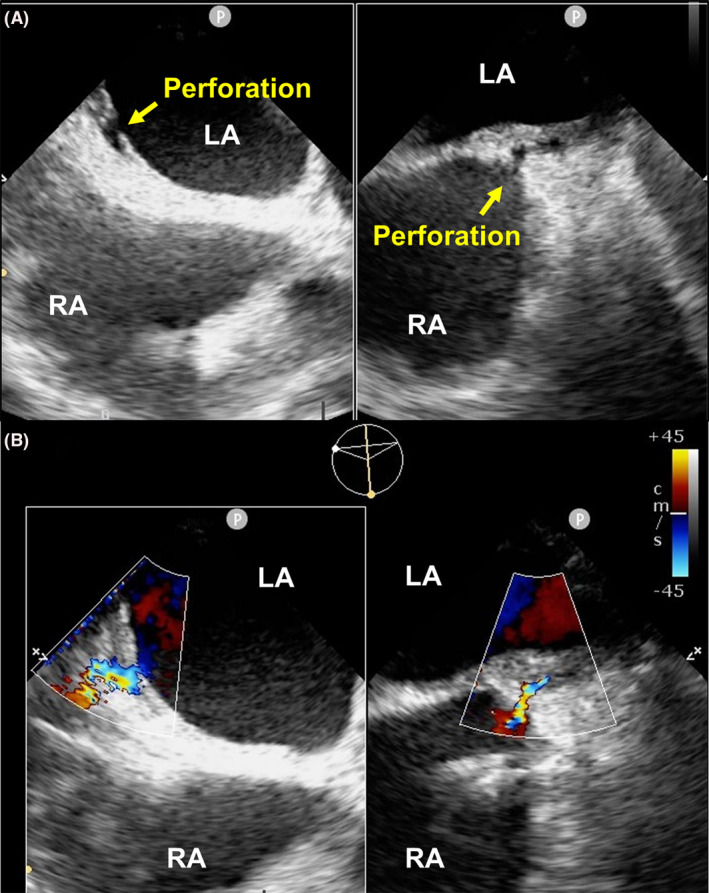
(A) A two‐dimensional image revealing the atrial septal perforation. (B) A color‐flow Doppler image revealing the atrial septal perforation. LA, left atrium; RA, right atrium

**FIGURE 4:**
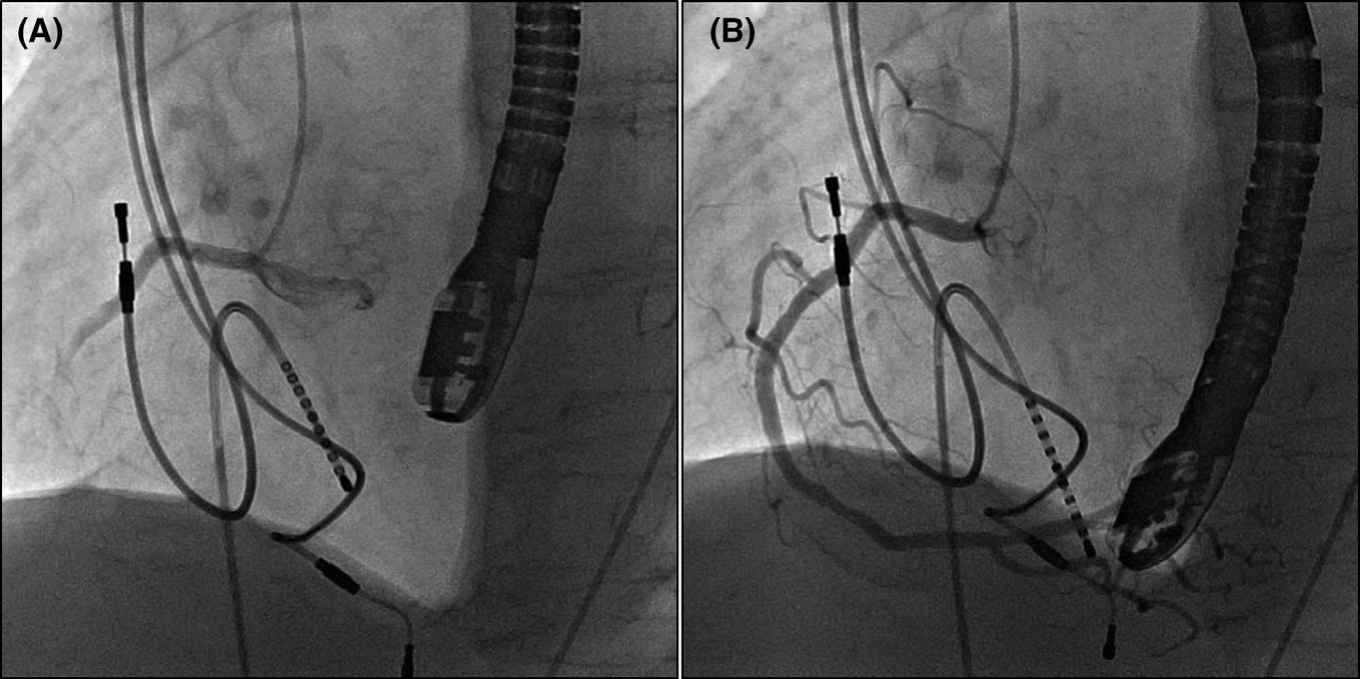
(A) The paradoxical air embolism in the right coronary artery. (B) Resolution of air embolism

An 82‐year‐old man with sick sinus syndrome was referred to our hospital with a pocket infection in the left pectoral region. Twenty‐nine years previously, he had been fitted with a dual‐chamber permanent pacemaker with two leads. Transvenous lead extraction was performed in a hybrid operating room under general anesthesia by dedicated cardiologists and cardiac surgeons, with extracorporeal circulation on standby. The infected pocket was widened and heavily debrided of all inflammatory tissue. The leads were dissected from the adhesions inside the pocket, and the fixation sutures were removed. We attempted to implement a combined superior and femoral approach to achieve appropriate co‐axial alignment of the powered sheath with the targeted leads because an extraction using the superior approach alone would have been difficult since the leads were old. Before starting the superior approach, an 18‐Fr sheath was introduced into the femoral vein, and the femoral approach was attempted using the Needle's Eye Snare. This involved the snare being twisted around the targeted lead using a clockwise or counterclockwise rotation of the snare, initially with a small retriever (13 mm) and then with a larger one (20 mm) (Figure 1). Despite several attempts, the lead was never grasped, and during this period, the patient's blood pressure suddenly decreased from 120/70 to 80/50 mm Hg accompanied by ST‐segment elevation that was detected using continuous bedside electrocardiogram monitoring. Transesophageal echocardiography revealed air bubbles (Figure 2 and Video S1) and atrial septal perforation (Figure 3 and Videos S2 and S3). Coronary angiography revealed a right coronary artery air embolism, which was resolved by repeated saline flushes that normalized the blood pressure and ST‐segment elevation (Figure 4 and Videos S4 and S5). The risk of a systemic air embolism seemed small, provided the femoral approach using the Needle's Eye Snare was discontinued; therefore, the femoral approach was abandoned. Thereafter, the superior approach using a powered sheath was utilized to successfully extract both leads. The patient was then discharged from the hospital in good health without any requirement for further procedures.

## 3 DISCUSSION

To the best of our knowledge, this is the first case of an atrial septal perforation associated with the Needle's Eye Snare. This case demonstrates the importance of not persisting unnecessarily with the use of the Needle's Eye Snare to prevent unnecessary atrial damage, as an excessive number of attempts may cause atrial injury. This is because the atrial septal perforation may have been caused by the manipulation of the Needle's Eye Snare. Namely, rotating the needle's eye and extending the threader too many times might have damaged the atrial septum and caused the perforation. Additionally, a larger Needle's Eye Snare could have presented a greater risk of causing atrial wall injury than a smaller one. Therefore, alternative strategies could have been considered, such as the use of an Agilis catheter (Abbot) in conjunction with an Ensnare device and a guidewire. When a guidewire placed through an Agilis catheter is wrapped around a lead by catching it with an Ensnare device, which establishes guidewire externalization, simultaneous downward traction of the guidewire and Ensnare device will result in the caudal pulling of the lead, and this will assist a powered sheath to advance over the lead via the subclavian vein. Moreover, a Goose Neck snare (Medtronic) or a lasso‐type snare catheter (Osypka) could be used with the femoral approach, if the lead has a free end.

Finally, particular attention should be paid to flushing the femoral sheath when exchanging the snares because air bubbles, which may result in a coronary embolism, are presumed to originate from the femoral sheath. More precisely, the interchange of different snares may introduce air bubbles into the femoral sheath.

## CONFLICT OF INTEREST

Authors declare no conflict of interest for this article.

## INFORMED CONSENT

Informed consent was obtained from the patient in this study.

## DISCLOSURE

The clinical description of this case report was approved by the Institutional Review Board of Sendai Kousei Hospital.

## Supporting information

Video S1

Video S2

Video S3

Video S4

Video S5

